# Commercial hydrogel product for drug delivery based on route of administration

**DOI:** 10.3389/fchem.2024.1336717

**Published:** 2024-02-27

**Authors:** Amin Raeisi, Fatemeh Farjadian

**Affiliations:** ^1^ Pharmaceutical Sciences Research Center, School of Pharmacy, Shiraz University of Medical Sciences, Shiraz, Iran; ^2^ Department of Pharmaceutics, School of Pharmacy, Shiraz University of Medical Sciences, Shiraz, Iran

**Keywords:** hydrogel, drug delivery, commercial products, routes of administration, buccal, transdermal, vaginal, oral

## Abstract

Hydrogels are hydrophilic, three-dimensional, cross-linked polymers that absorb significant amounts of biological fluids or water. Hydrogels possess several favorable properties, including flexibility, stimulus-responsiveness, versatility, and structural composition. They can be categorized according to their sources, synthesis route, response to stimulus, and application. Controlling the cross-link density matrix and the hydrogels’ attraction to water while they’re swelling makes it easy to change their porous structure, which makes them ideal for drug delivery. Hydrogel in drug delivery can be achieved by various routes involving injectable, oral, buccal, vaginal, ocular, and transdermal administration routes. The hydrogel market is expected to grow from its 2019 valuation of USD 22.1 billion to USD 31.4 billion by 2027. Commercial hydrogels are helpful for various drug delivery applications, such as transdermal patches with controlled release characteristics, stimuli-responsive hydrogels for oral administration, and localized delivery via parenteral means. Here, we are mainly focused on the commercial hydrogel products used for drug delivery based on the described route of administration.

## 1 Introduction

From scientific laboratories to clinical applications, the rise of commercial hydrogels has received significant attention in the field of biological advancements. The term “hydrogel” was first used by Van n Bemmelen in 1894. Later, in 1960, Lim and Wichterle demonstrated that hydrogels composed of poly (2-hydroxyethyl methacrylate) had potential applications as a filler after enucleation of the eye, fabrication of contact lenses, drug carriers, and arteries. As researchers delve deeper into the remarkable properties of these hydrophilic networks, their potential to revolutionize diverse biological applications becomes increasingly evident (van Bemmelen, 1894; Wichterle and LÍM, 1960; Peppas et al., 2006).

Hydrogels are complex networks of hydrophilic polymers that form three-dimensional (3-D) structures, enabling them to absorb and maintain significant volumes of biological fluids or water while preserving the integrity of their structure (Morteza et al., 2016). This balance relies on various factors, such as the selection of polymer types, the response to pH changes, cross-link density, and the behavior displayed in biological environments (Ullah et al., 2015). Functional polymer gels can swell because they have hydrophilic functional groups attached to polymer chains and cross-links between the chains (Douglas, 2018; Tanpichai et al., 2022). Hydrophilic functional groups, such as sulfates, carboxylic acids, hydroxyl groups, and amides, enable hydrogels to retain water and exhibit swelling behavior (Kesharwani et al., 2021).

Hydrogels offer remarkable versatility and are utilized in different subjects owing to their unique structures and compatibility with varying conditions of use (Morteza et al., 2016). Hydrogels show outstanding biomimetic properties because of multifunctional qualities, such as flexibility, softness, nontoxicity, biocompatibility, and biodegradability. These properties enable numerous biomedical, pharmaceutical, and other biomedical applications (Caló and Khutoryanskiy, 2015; Ahmad et al., 2019; Volpi et al., 2022). The particular physical characteristics of hydrogels have generated considerable investigation into their prospective uses in drug delivery applications. These biocompatible hydrogels have very advantageous physical characteristics, such as elasticity, that provide controlled release and long-term protection for the encapsulated entities (Gull et al., 2020). The porous structure of hydrogels can be easily modified by controlling the density of cross-links within the gel matrix and the hydrogels’ affinity for the external aqueous environment during swelling (Hoare and Kohane, 2008). The rapid diffusion of drug molecules into and out of swollen hydrogels, which involves drug entrapment and release, enables the utilization of cross-linked polymer networks in a hydrated or dehydrated state as effective carriers for drug delivery via various routes such as vaginal, ocular, oral, buccal, and parenteral administration (Erol et al., 2019).

The use of hydrogels in drug delivery systems has seen an increase in recent years. The current valuation of the hydrogel market stands at USD 22.1 billion as of 2019, with projected growth to reach USD 31.4 Billion by the year 2027. This growth is anticipated at a compound annual growth rate (CAGR) of 6.7% from 2020 to 2027. A rise in the utilization of hydrogel products stands as a significant stimulant for expanding the hydrogel market over the next few years (Cascone and Lamberti, 2020). Commercial hydrogels can be obtained for diverse drug delivery applications, including localized delivery through the parenteral route, stimuli-responsive hydrogels for oral delivery, and controlled release properties for transdermal patches (Shi and Li, 2005; Sharpe et al., 2014a; Zielińska et al., 2022).

**FIGURE 1:**
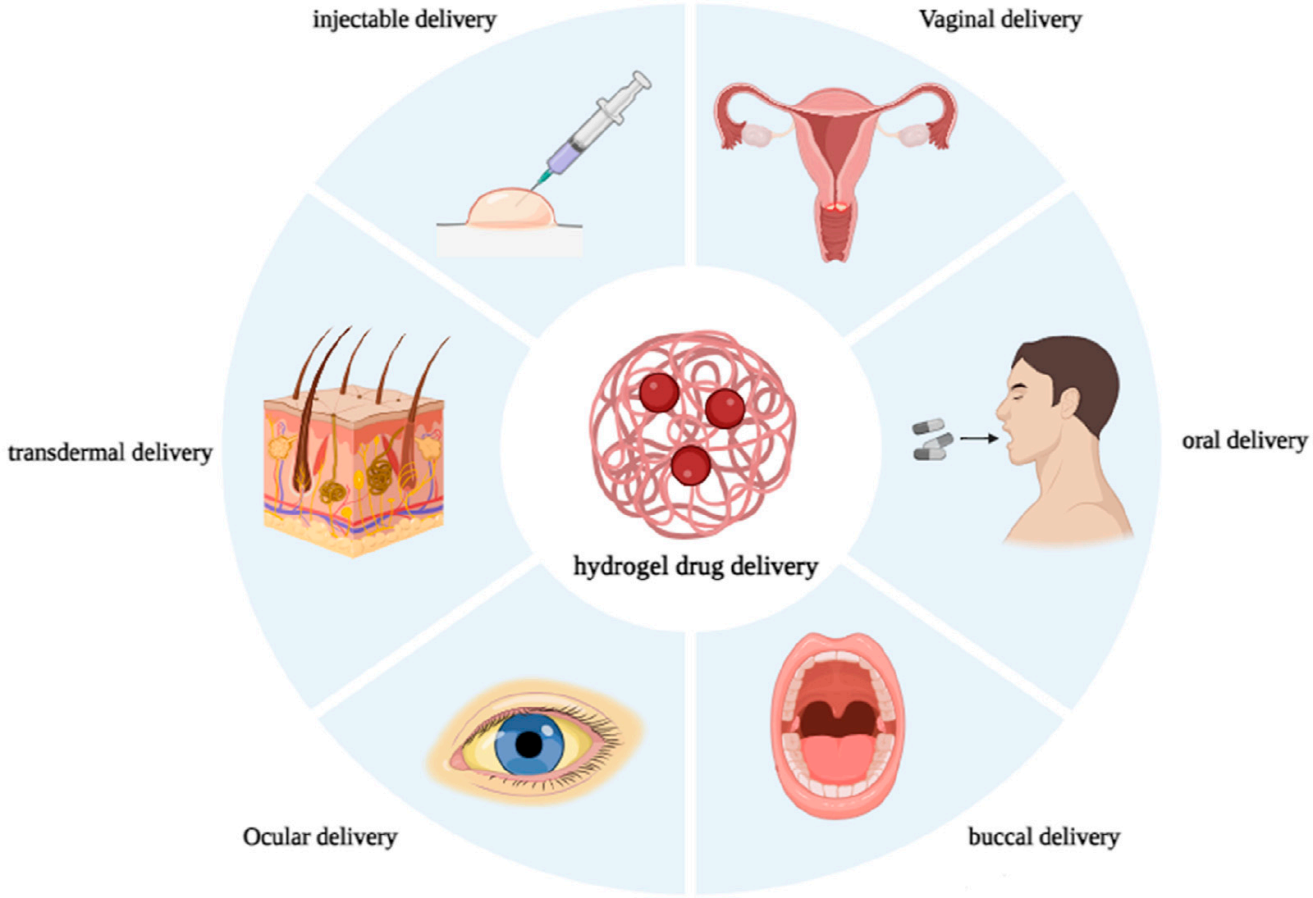
Application of hydrogel for drug delivery based on route of administration.

Research on hydrogel has been ongoing for many years, leading to the development of products that have found numerous applications in the drug delivery system. Therapeutic drugs are now loaded into polymer-based carriers, and the delivery and release of drug molecules is a topic of great interest in many medical fields (Roointan et al., 2018; Farjadian et al., 2019a; Farjadian et al., 2020; Farzanfar et al., 2021; Ghasemi et al., 2022a; Bahmani et al., 2022). The carriers facilitate the transportation of drugs to the specific target location (Entezar-Almahdi et al., 2021; Ghasemi et al., 2022b). Numerous studies have been published on commercial hydrogels for biomedical applications, specifically wound dressings, contact lenses, and cosmetics (Cascone and Lamberti, 2020). Following our previous reports on pharmaceutical applications of precious advanced materials (Farjadian et al., 2019b; Farjadian et al., 2019c; Ahmadi et al., 2020; Entezar-Almahdi et al., 2020; Hoseini-Ghahfarokhi et al., 2020; Zarkesh et al., 2021; Hejabi et al., 2022), in this paper we will discuss various commercial hydrogel products used for drug delivery, focusing on different routes of administration such as injectable, oral, buccal, ophthalmic, vaginal, and transdermal routes (Figure 1). Each section contains a table with an overview of the products on the market, their manufacturer, and their main ingredients.

## 2 Classification of hydrogel products

**FIGURE 2:**
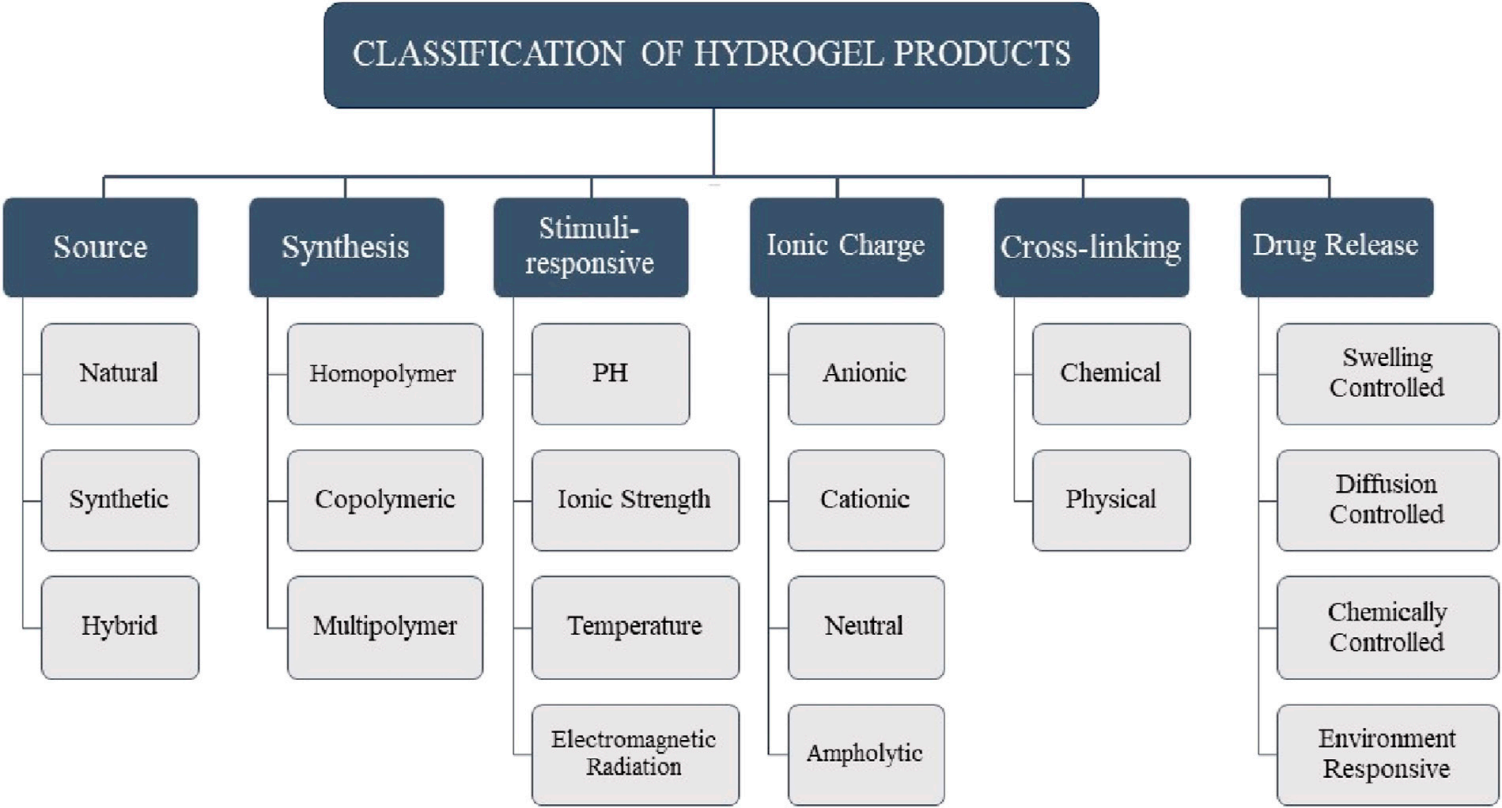
Classification of hydrogel products based on different parameters.

Hydrogels are cross-linked networks of hydrophilic polymeric chains. Hydrogels absorb about 70%–99% of their weight in water and can be used to formulate semi-solid delivery systems for labile and hydrophilic drugs (Narayanaswamy and Torchilin, 2020). Hydrogels can be classified in multiple ways (Figure 2). Based on their source, hydrogels are categorized as follows: natural hydrogels are biodegradable and biocompatible; synthetic hydrogels are non-toxic and compatible; and hybrid hydrogels, which combine synthetic and natural polymers for use in biomedical applications, are available (Malpure et al., 2018). Based on the synthesis route, hydrogels can be categorized into homopolymers, copolymers, and multipolymers. Hydrogels can exhibit different charge characteristics on the bound groups, leading to their classification as cationic, anionic, ampholytic, or electrically neutral, depending on the ionic charges present (Mahinroosta et al., 2018). Hydrogels, as complex polymeric structures, can display swelling behavior in response to various external stimuli. These stimuli can include alterations in pH, temperature, electromagnetic radiation, ionic strength, and other similar factors (Farjadian et al., 2023). The swelling of a polymeric chain occurs due to the hydration of hydrophilic and polar groups. The polar moieties expand, exposing hydrophobic groups that interact with water molecules. The polymeric network absorbs more water through an osmotic force, leading to infinite dilution (Holback et al., 2011). Hydrogels, according to the type of cross-linking, can be classified into two distinct categories: chemically cross-linked networks, characterized by the presence of permanent junctions, and physical networks, characterized by the formation of temporary junctions through polymer chain entanglements or physical interactions (Malpure et al., 2018). From the initial studies on the pharmaceutical applications of hydrogels, an extensive range of drug delivery systems were designed to prolong therapeutic efficacy and accomplish targeted delivery to particular tissues or organs. Hydrogels are divided into four groups concerning the mechanism controlling the drug release: 1) swelling-controlled, 2) diffusion-controlled, 3) chemically-controlled, and 4) environment-responsive systems (Mohite and Adhav, 2017).

## 3 Drug delivery commercial product

Hydrogels are frequently utilized as drug delivery vehicles. The commercial hydrogel-based drug delivery products are discussed in the following section according to the administration route.

### 3.1 Injectable drug delivery product

There has been a growing focus on injectable hydrogels compared to conventional gels in recent years. This is mainly due to their minimally invasive nature during surgery and ability to change shape in real time (Tan and Marra, 2010). Injectable hydrogels can be implanted through a minimally invasive procedure, significantly reducing patient discomfort and pain and decreasing healing time. Furthermore, this approach is cost-effective and applicable to hard-to-reach tissue sites (Rizzo and Kehr, 2021). Injectable hydrogels with multiple functions can be utilized to treat cancer, diabetes, and gene therapy by enabling the effective delivery of numerous pharmaceuticals and other substances, including proteins (Huang and Brazel, 2001). The main types of injectable hydrogels are synthesized by chemical or physical cross-linking.

**FIGURE 3:**
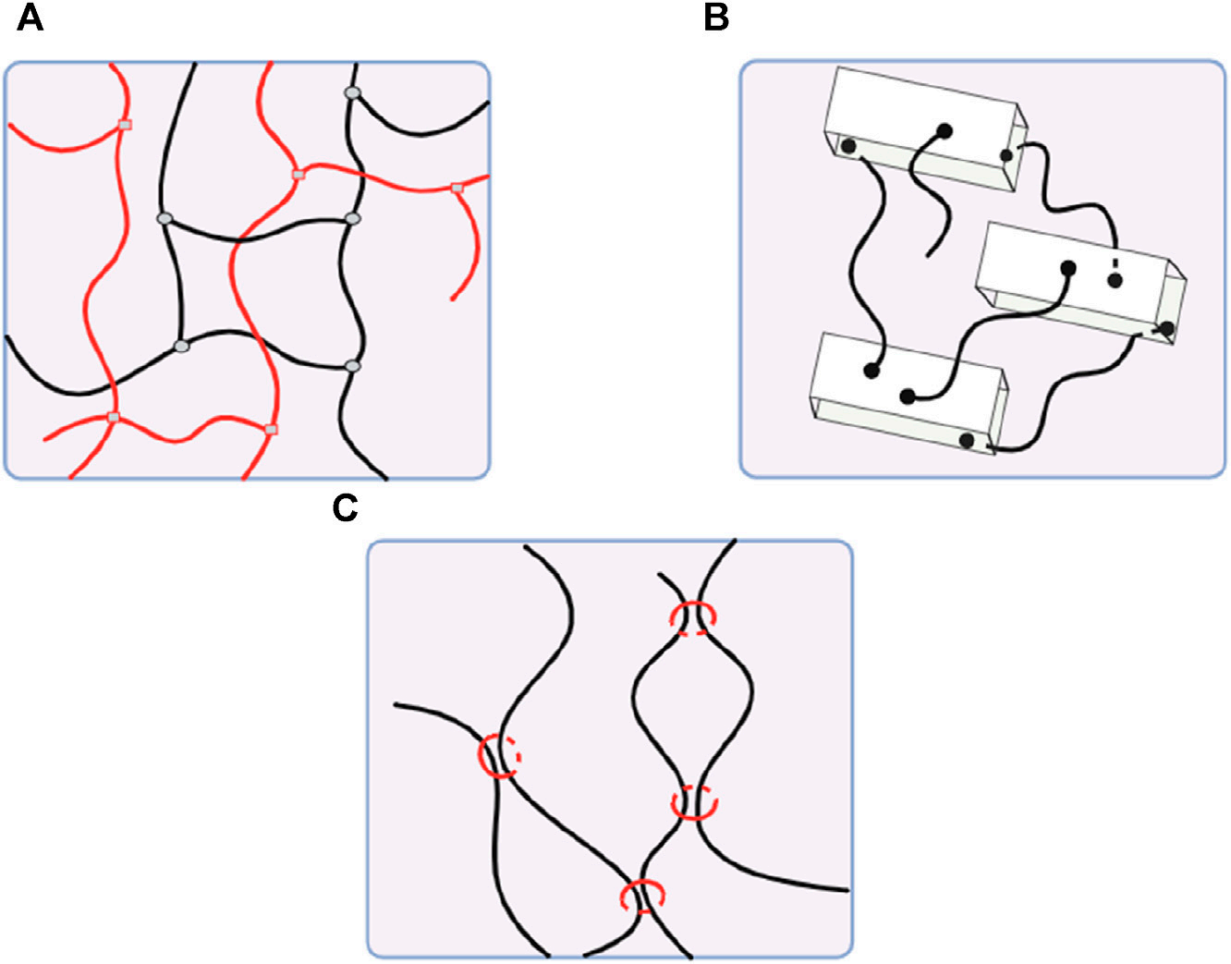
**(A)** Dual cross-linking hydrogel. **(B)** Nanocomposite hydrogel. **(C)** Slide-ring hydrogel.

Physically cross-linked hydrogels, developed by non-covalent secondary forces, are preferable for a sustained delivery system. In contrast, injectable hydrogels created by chemical cross-linking offer superior long-term stability and mechanical qualities but may cause adverse effects due to toxic crosslinkers (Yu and Ding, 2008; Mathew et al., 2018). Novel types of hydrogels, like dual cross-linking hydrogels, nanocomposite hydrogels, and slide-ring hydrogels, are developed to enhance mechanical properties (Figure 3) (Katsuno et al., 2013; Zhao et al., 2016).

Temperature-responsive hydrogels are as attractive as physical gels because they can be customized for specific purposes. These materials possess a biologically compatible *in situ* setting process, allowing for the incorporation of cells, bioactive compounds, and even intricate 3-D structures (Han et al., 2018). Hydrogel has been extensively investigated for localized therapy, but few have been commercialized because of its complicated delivery system, difficulties in large-scale production, failure to pass clinical trials, and related side effects (Mishra and Singh, 2021). Table 1 contains a list of several commercial products.

**TABLE 1:** Some of the injectable commercial hydrogels.

Product	Company	Main constituent	Drug	Application	Ref
SpaceOAR™	Boston Scientific	Polyethylene Glycol	-	Prostate Cancer	Mishra and Singh (2021)
OncoGel^®^	Protherics, inc	Poly (lactic-co-glycolic acid) and polyethylene glycol	Paclitaxel	Localized delivery of solid tumors	Gok et al. (2009), Hobzova et al. (2019), Guo et al. (2020)
Ozurdex^®^ implant	Allergan inc	Poly (lactic-co-glycolic acid)	Dexamethasone	Inflammation and suppressing immune responses	Kropp et al. (2014), Bahmanpour et al. (2023)
Retisert^®^	Bausch and Lomb	Polyvinyl alcohol	Fluocinolone acetonide	Inflammation, suppressing immune responses	Bahmanpour et al. (2023), Mishra et al. (2023)
Iluvien^®^	Alimera Sciences	Polyvinyl alcohol and silicone adhesives	Fluocinolone acetonide	Diabetic macular edema	Cascone and Lamberti (2020)
Eligard^®^	Tolmar Pharmaceuticals, inc	Use Atrigel^®^ system consists of a lactide-glycolide liquid polymer in one syringe and a lyophilized drug in a second syringe	Leuprolide acetate	Prostate cancer	Perez-Marrero and Tyler (2004)
Lupron Depot^®^	Takeda pharmaceuticals, Abbvie Endocrine inc	Poly (lactic-co-glycolic acid)	Leuprolide acetate	Prostate Cancer	Jain et al. (2016)

### 3.2 Oral drug delivery product

**FIGURE 4:**
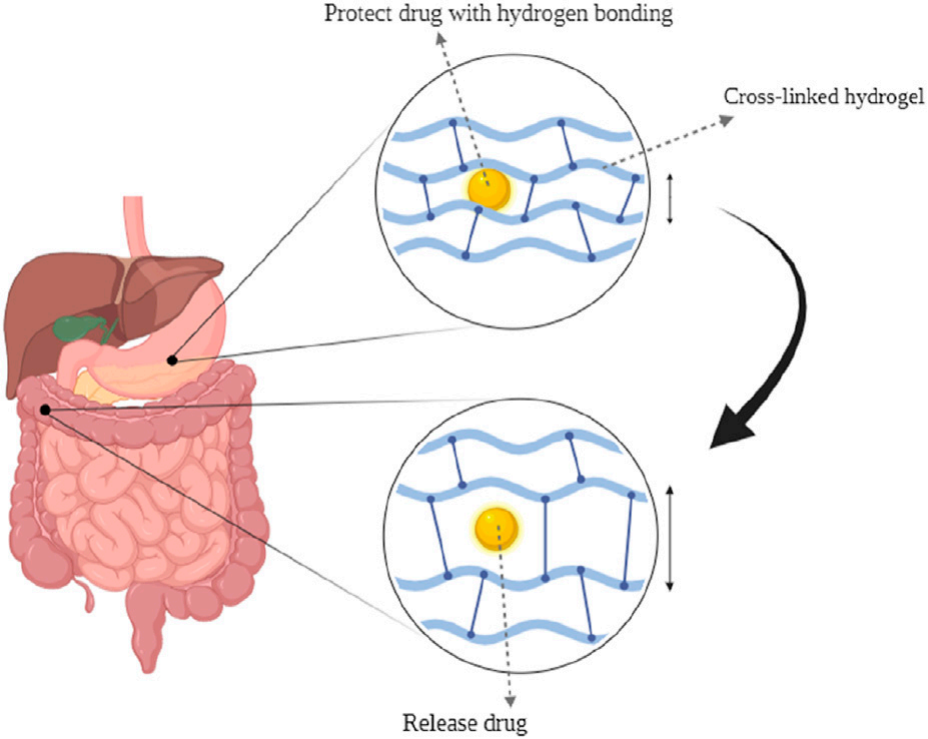
(1) preserving the drug in a low-pH environment. (2) Hydrogen bonds between polymer chains cause the carrier to become complex.

The oral route is a popular drug delivery method owing to its numerous benefits, including sustained and controlled drug delivery, ease of administration, feasibility of solid formulation, and high level of patient compliance (Homayun et al., 2019). The gastrointestinal tract (GIT) has a complex structure and functions that notably impact the release, dissolution, and absorption of orally administered dosage forms. This impact is primarily attributed to factors such as enzyme content, bile salts, acidity, and the mucosal absorptive surface of the GIT (Shah et al., 2017). Hydrogels are employed in smart oral delivery systems for hydrophobic biological molecules, facilitating site-specific dispersion of medicinal compounds within the intricate GIT. Traditional methods encounter obstacles such as limited permeability of the epithelial membrane and denaturation of drugs (Carr et al., 2010). Stimuli-responsive hydrogels are vital in oral delivery as they can respond to environmental changes. These hydrogels can be triggered by various physical and chemical stimuli, like pH, light, ionic strength, solvent composition, temperature, and electric field (Peppas et al., 2000; Farjadian et al., 2022). Drug delivery systems can be designed to administer therapeutic substances to specific organs in a regulated and predictable manner (Gull et al., 2019a). In the complicated environment of the GI tract, hydrogels protect therapeutic agents and allow targeted delivery by taking advantage of basic physiological changes (Figure 4) (Sharpe et al., 2014b). There have been studies of controlled release systems used in various pH ranges within the body, including the oral, intestines, gastric, and periodontal areas (Gull et al., 2019b). Hydrogels were extensively studied for their potential application in oral drug delivery of insulin, trying to solve the challenges associated with parenteral insulin administration. However, despite numerous advances in this field, progress has been limited, and there is now no commercially successful oral insulin product available for human use (Chaturvedi et al., 2013). Biopolymers such as alginate, hydroxypropyl cellulose, soybean protein, pectin, and cellulose acetate have been the subject of significant investigation in the field of GIT drug delivery (Vashist et al., 2014). Researchers are now working on the development of cost-effective and efficient drug delivery methods utilizing hydrogel materials. Numerous hydrogel systems have been designed to achieve controlled drug release through the utilization of various mechanisms that arise during the administration of medications through the oral route. Despite the challenges encountered by oral drug delivery, these systems have been designed to successfully solve these obstacles (Farrukh et al., 2023). Some examples of these commercial products are included in Table 2.

**TABLE 2:** Some of the commercial oral hydrogels.

Product	Company	Main constituent	Drug	Application	Ref
Voltaren^®^	GSK plc	Hydroxypropyl methylcellulose and polyethylene glycol	Diclofenac sodium	Anti-inflammatory	Cascone and Lamberti (2020)
Vicoprofen^®^	Abbvie ltd	Hydroxypropyl methylcellulose	Ibuprofen and hydrocodone bitartrate	Severe pain	Cascone and Lamberti (2020)
Levora^®^	Mayne pharma inc	Croscarmellose sodium	Levonorgestrel and ethinyl estradiol	Contraceptives	Cascone and Lamberti (2020)
Suprax^®^	Sanofi Aventis	Hydroxypropyl methylcellulose	Cefixime	Antibiotic	Cascone and Lamberti (2020)
Lopid^®^	Pfizer Inc	Hydroxypropyl methylcellulose	Gemfibrozil	Antibiotic	Cascone and Lamberti (2020)
Cytotec^®^	Pfizer	Hydroxypropyl methylcellulose	Misoprostol	Termination of pregnancy	Organization (2016)
Xartemis XR^®^	Mallinckrodt	Polyvinyl alcohol and hydroxypropyl cellulose	Oxycodone hydrochloride and acetaminophen	Acute pain	Jindal et al. (2022)
Inderal LA^®^	AstraZeneca	Ethylcellulose	Propranolol hydrochloride	Hypertension	Mu et al. (2003)
Zuplenz^®^	Aquestive Therapeutics	Polyethylene glycol 1000, polyvinyl alcohol, and rice starch	Ondansetron	Antiemetic	Jacob et al. (2021)
Theo-24^®^	Endo Pharmaceuticals, Inc	Hydroxypropyl methylcellulose	Theophylline anhydrous	Asthma and COPD	Wasilewska and Winnicka (2019)
Gaviscon^®^	Reckitt Benckiser Healthcare Ltd	carbomer 974p, Sodium alginate	Magnesium carbonate aluminum hydroxide	Antacid	Sudhakar et al. (2006)
Concerta^®^	Alza Corporation	Hydroxypropyl methylcellulose, poloxamer	Methylphenidate	Attention deficit hyperactivity disorder (ADHD)	Coskun et al. (2009)
Noxafil^®^	Merck	Hydroxypropyl methylcellulose	Posaconazole	Antifungal	Nyamweya (2021)

### 3.3 Buccal drug delivery product

**FIGURE 5:**
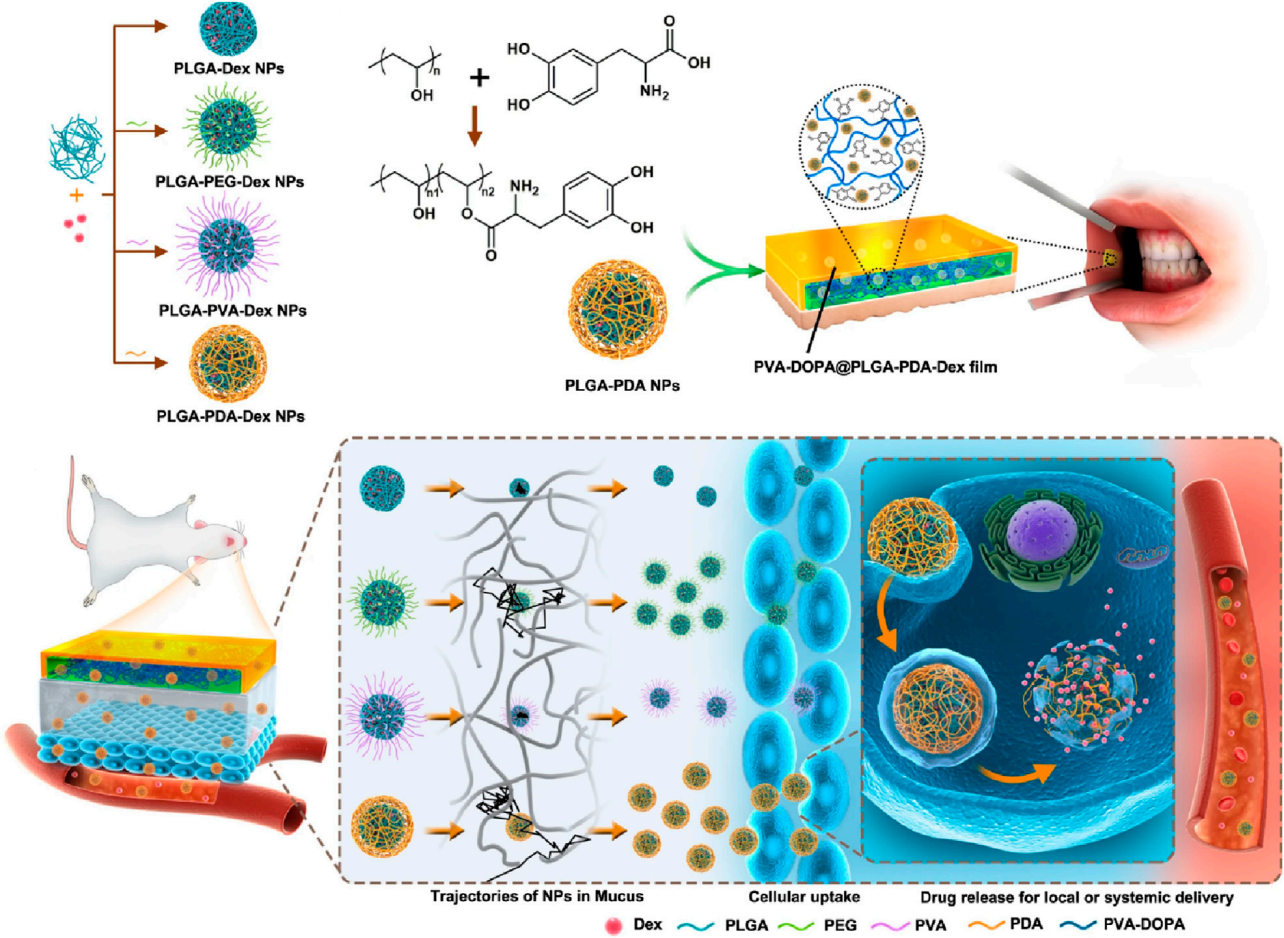
The synthesis and biomedical application of PVA-DOPA@NPs-Dex mucoadhesive film enhances mucoadhesion for buccal drug delivery. PVA: poly (vinyl alcohol), DOPA: 3,4-dihydroxy-D-phenylalanine, NPs: nanoparticles, Dex: dexamethasone. Adopted from (Hu et al., 2021).

The buccal mucosa is highly accessible and characterized by a wide area of smooth muscle and generally static mucosa, making it an ideal site for administering retentive dosage forms. The internal jugular vein bypasses the hepatic first-pass metabolic process to allow direct entry of medicines into the systemic circulation, resulting in increased bioavailability. Additional benefits include minimal enzymatic activity, compatibility with medications or excipients that cause only minor and reversible mucosal damage or irritation, administration without pain, and convenient drug withdrawal (Sudhakar et al., 2006). Drug administration through the buccal route provides a viable alternative for drug delivery to the systemic circulation. In the study of Hu *et al.*, a mucoadhesive film inspired by mussels and containing polydopamine (DOPA) nanoparticles has been shown to have more significant advantages for transporting drugs over the mucosal barrier, as well as increased drug bioavailability and therapeutic efficacy in models of oral mucositis (Hu et al., 2021). The synthesis of PVA-DOPA polymers involved the modification of poly (vinyl alcohol) (PVA) using the mussel adhesive protein DOPA. Then, different PLGA (poly (D,L-lactide-co-glycolide) nanoparticles were integrated into the PVA-DOPA film to create a combined buccal drug delivery system with dexamethasone (Dex) known as the PVA-DOPA@NPs-Dex film **(** Figure 5). The nanoparticles are slowly released from the film, then move through the mucus layer and pass through the epithelium. This results in sustained drug release and increased therapeutic efficacy in the treatment of oral mucositis.

Various commercial buccal medication administration dosage forms are available on the market, including buccal tablets, sprays, mucoadhesive formulations, sublingual lozenges, chewing gums, films, and solutions (Nagai and Machida, 1993). There are numerous disadvantages associated with buccal drug delivery. Firstly, the buccal mucosa acts like a barrier that may limit the permeability of certain drugs. Secondly, the presence of saliva can potentially dilute the drug, thereby affecting its absorption. Lastly, the oral cavity experiences a variable environment due to factors such as food consumption and other daily activities. To address this challenge, many strategies can be employed for buccal drug delivery, including physical penetration enhancers like iontophoresis, chemical penetration enhancers like surfactants, and formulation technologies like polymeric and mucoadhesive dosage forms (Wanasathop et al., 2021). Their bioadhesive qualities significantly influence the choice of hydrogel delivery methods. When a hydrogel has high adhesion to the epithelium, it can extend the system’s retention at a target site, delivering enough drug doses for the intended therapeutic effect. This is especially crucial for buccal delivery (Peppas and Sahlin, 1996). Specific polymers, like poly (acrylic acid) and chitosan, have been observed to possess mucoadhesive properties. Poly (acrylic acid) can establish hydrogen bonds with the mucosa, whereas chitosan, which carries a positive charge, can engage in electrostatic interactions with negatively charged surfaces of tissues and cells (Li and Mooney, 2016). Hydrogel carriers for drug delivery contain the potential to control the release rate of drugs based on the hydration state. This hydration level determines the hydrogel’s swelling ability (Narayanaswamy and Torchilin, 2020). In the field of drug delivery, a variety of material-based hydrogels have been commercially applied. Some commonly used hydrogels include hydroxyethyl cellulose, hydroxypropyl cellulose, polyvinyl alcohol, polyacrylic resins, carboxymethyl cellulose, and hydroxypropyl methylcellulose (Cascone and Lamberti, 2020). Some of these commercial products are listed in Table 3.

**TABLE 3:** Some of the transdermal commercial hydrogels.

Formulation	Product	Company	Main constituent	Drug	Application	Ref
Buccal tablet	Imdur^®^	Key pharmaceuticals	Hydroxypropyl cellulose and Hydroxypropyl methylcellulose	Isosorbide mononitrate	Prevention angina attacks	Cascone and Lamberti (2020)
	Nicorette^®^	Johnson&Johnson	Hydroxypropyl methylcellulose	Nicotine	Quit smoking	Cascone and Lamberti (2020)
	Suscard Buccal^®^	Pharmax Limited	Hydroxypropyl methylcellulose	Glyceryl trinitrate	Prevention angina attacks	Sudhakar et al. (2006)
	Striant^®^	Mipharm S.p.A	Carbomer and hydroxypropyl cellulose	Testosterone	Hormone replacement therapy	Cascone and Lamberti (2020)
	Aphtach^®^	Teijin Ltd	Hydroxypropyl cellulose and carbomer	Triamcinolone acetonide	Anti-inflammatory	Cascone and Lamberti (2020)
	Buccastem®M	Alliance Pharma	Xanthan gum	Prochlorperazine maleate	Antiemetic	Cascone and Lamberti (2020)
Buccal film	Zilactin^®^	Zila pharmaceuticals	Hydroxypropyl cellulose	Benzocaine	Pain	Sudhakar et al. (2006)
	BELBUCA^®^	Biodelivery Sciences	Hydroxyethyl cellulose	Buprenorphine	Moderate to severe pain	Nyamweya (2021)
Semisolid	Adcortyl in orabase^®^	Bristol-Myers Squibb	Pectin, gelatin, carboxymethylcellulose	Triamcinolone acetonide	Anti-inflammatory	Cascone and Lamberti (2020)
	Bioral gel^®^	Merck	Carboxymethyl cellulose	Carbenoxolone	Anti-Inflammatory	Cascone and Lamberti (2020)
Solution	Lubrajel™ BA	Ashland	Glyceryl polyacrylate and glyceryl acrylate	-	Oral moisturizing	Cascone and Lamberti (2020)

### 3.4 Ocular drug delivery product

**FIGURE 6:**
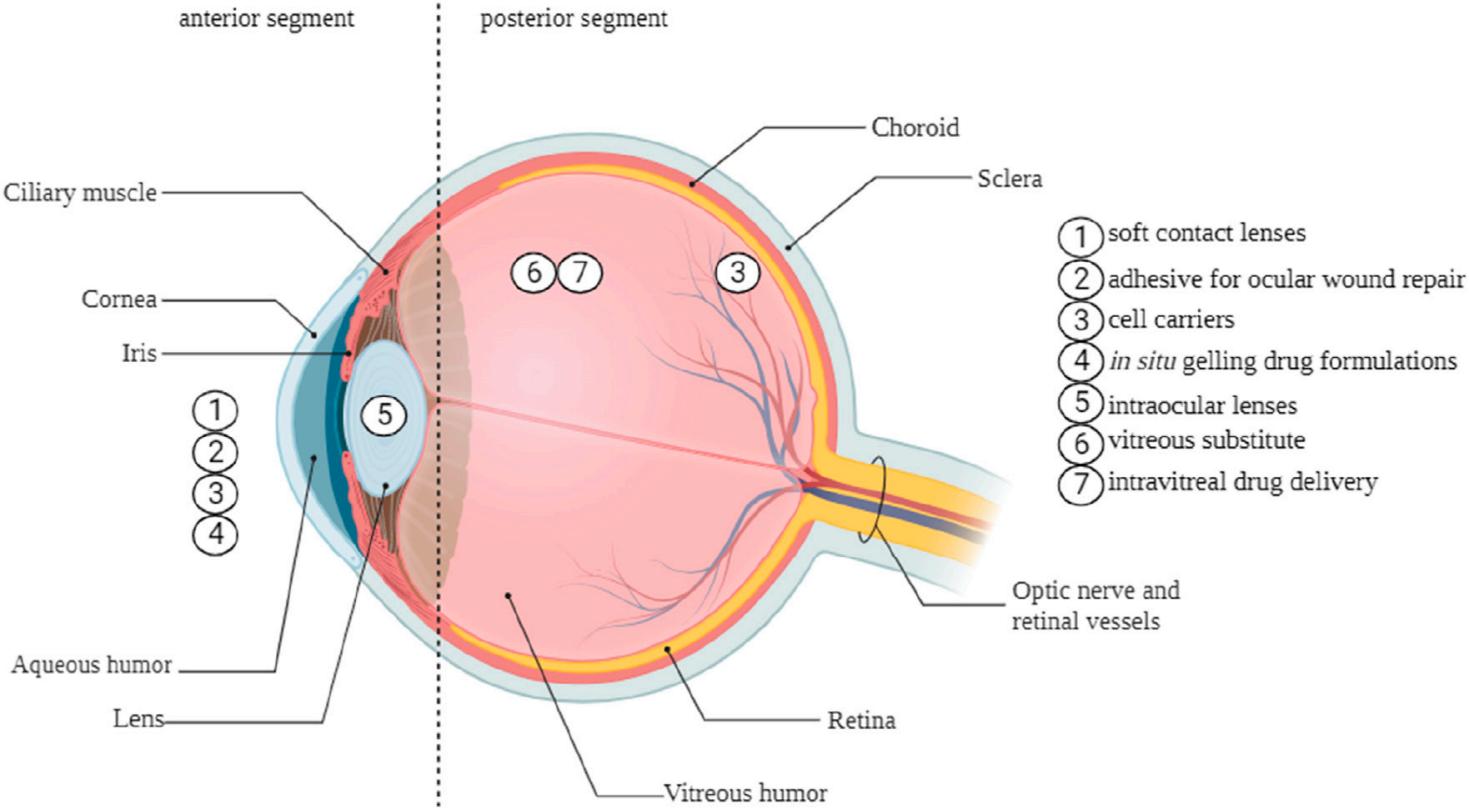
The application of hydrogels in various ocular regions.

The human eye is a complex, spherical organ. The structure can be anatomically separated into two compartments, namely, the anterior and posterior segments. Ophthalmic drugs administered to the anterior section of the eye encounter challenges from dynamic and static barriers. The main barriers are the blood-queous barrier, corneal epithelium, corneal stroma, lymph flow, conjunctival blood flow, and tear drainage. These factors play a critical role in the formulation and development of ophthalmic therapies (Torres-Luna et al., 2020). Drug contact with ocular surface tissues is brief, often lasting between 1 and 2 min. This is due to the continuous generation of tears, which range from 0.5 to 2.2 μL per minute, as well as the turnover of tears (Sánchez-López et al., 2017). Hydrogels possess the capability to effectively resist the rapid blinking and flushing actions of tears, thereby extending the duration of drug presence on the eye surface. This prolonged drug resident time facilitates enhanced drug efficacy regarding localized therapeutic action on mucosal surfaces or more profound penetration into eye tissues. Hydrogels can extend the duration of drug presence on the ocular surface and maintain a controlled release of drugs within the intraocular tissues, including the vitreous cavity and aqueous humor (Cooper and Yang, 2019). The versatility and modifiability of hydrogels make them ideal for efficiently transporting medications to the eye, as they may be designed to take advantage of the specific function and environment for which they are developed (Torres-Luna et al., 2020; MohammadSadeghi et al., 2021). Figure 6 illustrates the diverse range of potential applications of hydrogels in therapeutic ophthalmic.

Hydrogels can augment medication retention on the ocular surface by elevating solution viscosity, facilitating their swelling in water or aqueous solvents. Chitosan, hydroxypropyl cellulose, dextran, hydroxypropyl methylcellulose, and poly (acrylic acid) derivatives, including polycarbophil and carbomer 934, have been identified in studies that are most appropriate for bioadhesive polymers for ocular medication administration (Fathi et al., 2015). Table 4 is a compilation of several commercial products.

**TABLE 4:** Some of the ocular commercial hydrogels.

Product	Company	Main constituent	Drug	Application	Ref
Akten™^®^	Akten	Hydroxypropyl methylcellulose	Lidocaine	Ocular surface anesthesia	Chowhan and Giri (2020)
Liquivisc^®^	Laboratoires THEA	Carbomer 974P	-	Dry eye	Fang et al. (2021)
Hylo^®^ Gel	Candorpharm Inc	Sodium hyaluronate	-	Dry eye	Tomczak et al. (2021)
Viscotears^®^	Novartis	Carbomer 980	-	Dry eye	Fang et al. (2021)
Liposic^®^	Bausch + lomb	Carbomer 980	-	Dry eye	Kalhori et al. (2016)
Clinitas Gel^®^	Altacor	Carbomer 980	-	Dry eye	Fang et al. (2021)
ReSure®Sealant	Ocular Therapeutix	Polyethylene glycol	-	Seal clear corneal incisions following cataract surgery	Spierer and O'Brien (2015)
Timolol GFS^®^	Alcon	Gellan gum and xanthan gum	Timolol maleate	Glaucoma	Chowhan and Giri (2020)
Timoptic XE^®^	Merck	Gellan gum	Timolol maleate	Glaucoma	Wu et al. (2019)
Timoptol-LA^®^	Merck Sharp and Dohme	Gellan gum	Timolol maleate	Glaucoma	Wu et al. (2019)
Pilopine HS^®^	Alcon	Carbomer	Pilocarpine	Glaucoma	Al-Kinani et al. (2018)
Pilogel^®^	Alcon	Carbomer 940	Pilocarpine	Glaucoma	Al-Kinani et al. (2018)
Carteol LP^®^	Alcon	Alginic acid	Carteolol hydrochloride	Glaucoma	Chowhan and Giri (2020)
Aza Site^®^	In Site Vision	Hydroxypropyl methylcellulose (HPMC E15 LV and HPMC K4M)	Azithromycin	To treat bacterial infection	Chowhan and Giri (2020)
DuraSite^®^/Azasite^®^	Inspire Pharmaceuticals	Polycarbophil (carbomer cross-linked with divinyl glycol)	Azithromycin	To treat bacterial infection	Al-Kinani et al. (2018)
AzaSite plus^®^	Inspire Pharmaceuticals	Polycarbophil (carbomer cross-linked with divinyl glycol)	Azithromycin and dexamethasone	Anti-bacterial and anti-inflammation	Al-Kinani et al. (2018)
Virgan^®^	Thea Pharmaceuticals	Carbomer	Ganciclovir	antiviral	Al-Kinani et al. (2018)

### 3.5 Vaginal drug delivery product

The vagina is an essential reproductive organ made of a 7.50-cm-long muscular canal located between the urethra, rectum, and bladder. The vaginal membrane is composed of three different layers, including the muscular coat, epithelial layer, and tunica adventitia. The thickness of the vaginal epithelium is dependent upon various factors, including age, hormonal activity, and life phases. The vaginal branch of the uterine artery is responsible for providing blood circulation to the vagina (Valenta, 2005). The vaginal route of drug administration is thought to be highly advantageous owing to its extensive blood flow, capacity to elude the first-pass effect, and significant permeability to many pharmacological compounds, including peptides and proteins (Aka-Any-Grah et al., 2010). A variety of pharmacological groups, including antimicrobials, labor inducers, sexual hormones, and spermicides, have been delivered via the vaginal mucosa. The majority of these drugs are employed for localized conditions, while a few of them can achieve serum concentrations that are adequate for producing systemic effects (Alexander et al., 2004). Vaginal drug delivery methods use natural or synthetic polymers to facilitate drug interaction with the target site and achieve controlled, reproducible, and predictable drug release. Various systems are presently being utilized or under examination (Osmałek et al., 2021). The term “mucoadhesion” pertains to when a substance with biological origin is bound to the mucosa surface for a prolonged period due to interfacial forces (Das Neves and Bahia, 2006). The mucoadhesive system possesses the capacity to regulate the rate of drug release from the vaginal canal and prolong its residence time. Bioadhesion has the potential to enhance the level of contact and extend the residence time of dosage forms across different administration routes. These formulations have the potential to include pharmaceutical substances or function in combination with moisturizing agents to serve as a means of managing vaginal dryness (de Araujo Pereira and Bruschi, 2012). A variety of bioadhesive polymers were evaluated to establish a vaginal delivery system. These polymers included sodium alginate, polycarbophil, guar gum and xanthan, Carbopol^®^, sodium carboxymethylcellulose, hydroxypropyl methylcellulose, and hydroxypropyl cellulose (Veiga et al., 2018). Mucoadhesive vaginal drug delivery systems include gels, tablets, films, emulsion-type systems, and suppositories, but gels are the primary mucoadhesive vaginal drug delivery methods in current usage. Vaginal formulations are characterized by their ease of manufacturing, comfort, and efficacy in establishing intimate contact with the vaginal mucosa. These compounds contain a significant amount of water and exhibit rheological qualities, hence offering hydrating and lubricating effects, particularly in cases of dry vaginal mucosa. The utilization of mucoadhesive polymers has been shown to enhance the duration of contact, reduce formulation loss, and prolong the therapeutic impact (Das Neves and Bahia, 2006; de Araujo Pereira and Bruschi, 2012). Table 5 presents a comprehensive overview of several proprietary vaginal compositions now accessible in the marketplace.

**TABLE 5:** Some of the vaginal commercial hydrogels.

Product	Company	Main constituent	Drug	Application	Ref
Vagisil^®^	Combe, inc	Hyaluronic acid	-	Vaginal moisturizer	Cascone and Lamberti (2020)
K-Y^®^	Johnson and Johnson	Hydroxyethyl cellulose	-	Vaginal lubrication	Das Neves and Bahia (2006)
Replens^®^	Lds consumer	Carbopol^®^ 974p and polycarbophil	-	Vaginal moisturizer	Acarturk (2009)
Aci-Jel^®^	Hope pharmaceuticals	Acacia gum, tragacanth	Oxyquionoline sulfate, acetic acid, ricinoleic acid	Maintenance of the vaginal acidity, antiseptic	Das Neves and Bahia (2006)
Crinone^®^	Merck Serono	Carbopol^®^974p and polycarbophil	Progesterone	Infertility, secondary amenorrhea	Acarturk (2009)
Vagifem^®^	Novo Nordisk	Hypromellose	Estradiol	Atrophic vaginitis	Schulz et al. (2013)
Carraguard^®^	David M. Phillips Laboratory	Carrageenan PDR98-15	Progestin levonorgestrel	Contraceptive	Brache et al. (2009)
Cervidil^®^	Ferring Pharmaceuticals Inc	Polyethylene oxide	Dinoprostone	Start ripening of the cervix in pregnant women	Aswathy et al. (2020)
Conceptrol^®^	Advanced Care Products	Sodium carboxymethylcellulose	Nonoxynol-9	Contraceptive	Das Neves and Bahia (2006)
Advantage-S®^b^	Columbia laboratories	Polycarbophil, carbopol^®^ 974p	Nonoxynol-9	Contraceptive	Das Neves and Bahia (2006)
Gynol II^®^	Mcneil-PPC, Inc	Sodium carboxymethylcellulose	Nonoxynol-9	Contraceptive	Acarturk (2009)
Encare^®^	Thompson Medical Co. Inc	Polyethylene glycol	Nonoxynol-9	Contraceptive	Raymond et al. (2004)
Prostin E2^®^	Pfizer	Colloidal silicon dioxide	Dinoprostone	Labour inducer	Acarturk (2009)
Metrogel Vaginal^®^	Galderma (United Kingdom) Ltd	Carbopol 974p	Metronidazole	Bacterial vaginosis	Acarturk (2009)

### 3.6 Transdermal drug delivery product

The skin, which is the largest and most external organ within the human body, covers an approximate surface area of 1.8 square meters and constitutes approximately 20% of the whole body weight of an average individual (Brown and Williams, 2019). The utilization of the skin as a way of drug delivery presents advantages such as sustained release, greater compliance, and reduced rates of adverse effects in comparison to oral and parenteral administration methods. The transdermal delivery method is considered ideal due to its large surface area, minimal potential for enzyme-dependent degradation, and extended duration of application (Akbarian et al., 2022; Dahri et al., 2023). The stratum corneum offers an important challenge in the transdermal administration of active substances due to its role as the initial barrier of the skin. This barrier restricts drug absorption and limits the range of medications that can be effectively given (Al-Japairai et al., 2020). Polymers are of significant importance in the formulation of skin preparations since they act as a matrix for delivering the active ingredient to the desired application site or target organ. This intricate system distinguishes between active and inactive components, achieving the desired result. Polymers possess diverse applications, serving as gelating agents in gel systems, consistency excipients in emulsions and creams, matrix materials in patches, and skin adhesives in transdermal systems. Their diverse range of applications makes them useful in numerous formulations for skin preparations (Valenta and Auner, 2004). Hydrogels, which are derived from hydrophilic polymers, exhibit favorable characteristics for drug delivery purposes owing to their capacity to hold large amounts of water, thus enhancing skin elasticity and moisturization (Viyoch et al., 2005). Hydrogel-based patches offer the capacity to deliver drugs in a controlled manner over a specified duration, rendering them more favorable compared to conventional methods.

Additionally, these systems possess the advantage of being quickly packaged and transported while also enabling specific drug delivery to the intended site (Boriwanwattanarak et al., 2008). The Scopolamine patch, which represents the first marketed transdermal delivery patch, presents a revolutionary approach to preventing the effects of motion sickness (Prausnitz et al., 2012). Transdermal patches are available in various types, such as single-layer, multi-layer, reservoir, matrix, and vapor patches. In a single-layer patch, the adhesive layer contains and releases the drug. The multi-layer patch consists of two distinct layers designed for immediate and controlled drug release, which are effectively separated by a membrane. The reservoir transdermal system includes different layers, including a drug layer and a liquid compartment containing a drug suspension or solution, separated by an adhesive layer. The release rate follows zero-order kinetics (Pastore et al., 2015). The matrix system consists of a partially overlaid adhesive layer and a semi-solid matrix, which includes a drug solution or suspension. The novel-type vapor patch is designed to deliver essential oils continuously for over 6 h. Its primary purposes include decongestion and enhancing sleep quality (Lee et al., 2001). Hydrogels are utilized for transdermal delivery in patches or creams, as they effectively enhance drug permeation by promoting skin hydration.

Moreover, hydrogels are well-suited for topical applications. Furthermore, investigations have been conducted on their potential to improve the stability and efficacy of transdermal delivery systems such as liposomes, micelles, and nanoparticles (Kong et al., 2016). Table 6 presents examples of commercially available transdermal hydrogels for drug delivery.

**TABLE 6:** Some of the transdermal commercial hydrogels [data from the Food and Drug Administration (FDA)].

Product	Company	Main constituent	Drug	Application	Ref
Lidoderm^®^	Endo Pharmaceuticals Inc	Glycerin, polyacrylic acid, and polyvinyl alcohol	lidocaine patch 5%	post-herpetic neuralgia	Hydrogel Market (2020)
Nitro-Dur^®^	Merck and Co. Inc	Acrylic acid	Nitroglycerin	Angina pectoris	NITRO-DUR (2012)
Duragesic^®^	Janssen Pharmaceuticals	ethylene-vinyl acetate and hydroxyethyl cellulose	Fentanyl	Chronic pain	DURAGESIC (2021)
Daytrana^®^	Noven Pharmaceuticals	Acrylic and silicone adhesive	Methylphenidate	Attention deficit hyperactivity disorder	DAYTRANA (2010)
EMSAM^®^	Somerset Pharmaceuticals	Acrylic acid and ethylene vinyl acetate	selegiline	Major depressive disorder	EMSAM (2007)
Exelon^®^	Novartis Pharmaceuticals	Acrylic	Rivastigmine tartrat	Dementia	Exelon Patch (2007)
Estraderm^®^	Novartis Pharmaceuticals	hydroxypropyl cellulose	Estradiol	Hormone replacement therapy	Estraderm (2012)
Butrans^®^	Purdue Pharma	polyacrylate cross-linked with aluminium	Buprenorphine	Chronic pain	BUTRANS (2014)
Flector^®^	IBSA Farmaceutici Italia	Gelatine and propylene glycol	diclofenac epolamine	inflammation and pain	FLECTOR PATCH (2011)
Qutenza^®^	Averitas Pharma	ethyl cellulose and silicone adhesive	Capsaicin	Neuropathic pain	QUTENZA (2020)

## 4 Advantages and disadvantages

Hydrogels provide numerous advantages that make them ideal for biomedical applications, particularly drug delivery. These benefits include sustained action, decreasing administration doses, ease of modification, reduced side effects, drug targeting capabilities to specific locations, and the ability to respond to stimuli. Nevertheless, hydrogels exhibit several disadvantages, including hypoxia, dehydration, as well as limited mechanical strength, challenging manipulability, and high cost.

Oxygen is a vital factor in the existence of cellular organisms. Some hydrogels can induce hypoxia as a state of lack of oxygen. This phenomenon can be considered as a limitation or advantage of hydrogel application in therapy. Hypoxia-induced hydrogels in surrounding tissues can stimulate the invasion of blood vessels and activate hypoxia-regulated pathways to regenerate tissue in neovascularization (Park and Gerecht, 2014). One drawback of hydrogel utilization is dehydration, which can cause stiffening and locking their dynamic behavior. This can be observed in thermos-responsive hydrogel after reaching LCST and can be considered as a limiting factor in their application in therapy. (Zhang et al., 2023). Hydrogels have low mechanical strength due to two characteristics: high solution and low friction between chains (Lin et al., 2022). This problem has arisen by designing composite structures by integrating heterogeneous elements such as silica in the hydrogel structure (Xu et al., 2022). Overall, the advantages of hydrogels exceed their disadvantages in the field of drug delivery. Considering positive factors, hydrogel commercial administration in drug delivery will be vast.

## 5 Conclusion and future perspectives

Drug carriers represent revolutionary delivery systems within the science of medicine. Numerous studies have shown various polymers employed in carrier synthesis. Among these, hydrogel-based systems received significant attention due to their cost-effectiveness, ease of production, and remarkable capacity to carry diverse drug types. Utilizing cross-linked polymeric networks for enhancing therapeutic efficacy presents a novel way for drug delivery applications. Still, despite the numerous capabilities and advantages of hydrogels in the field of drug delivery, there remains an essential requirement for further research and development to efficiently and swiftly introduce more hydrogel-based formulations to the market. Novel features of hydrogels will continue to play a crucial function in drug delivery and enable the development of a vast array of drugs, peptides, proteins, and delivery systems.

Commercial hydrogel products for drug delivery based on different routes of administration represent a rapidly growing market with significant potential. Continued research and development in hydrogels hold promising prospects for the future. Advancements in drug delivery and polymer-based carrier systems will facilitate targeted and efficient transportation of therapeutic drugs to specific locations within the body. Continued research, technological innovations, and collaborative efforts can fully realize the potential of hydrogel drug delivery in improving therapeutic outcomes.
